# The utility of coded very high frequency telemetry for monitoring reintroduced mammal populations

**DOI:** 10.1002/ece3.10193

**Published:** 2023-06-15

**Authors:** Chloe H. Frick, Donell Hole, Derek Sandow, Liberty G. M. Olds, Bertram Ostendorf, David Taggart, David Roshier

**Affiliations:** ^1^ School of Biological Science University of Adelaide Adelaide South Australia Australia; ^2^ Lotek NZ Ltd Havelock North New Zealand; ^3^ Northern and Yorke Landscape Board Clare South Australia Australia; ^4^ Zoos South Australia Adelaide South Australia Australia; ^5^ FAUNA Research Alliance Kahibah New South Wales Australia; ^6^ School of Animal and Veterinary Science University of Adelaide Roseworthy South Australia Australia

**Keywords:** bettong, monitoring, radio tracking, reintroductions, VHF telemetry

## Abstract

Very high frequency (VHF) radio tracking technology deployed on terrestrial vertebrates has been well utilized in ecology without much evolution since the 1960s. With the advent of multi‐species rewilding projects, and the new field of reintroduction biology, there has been an increase in requirements for telemetry systems to monitor survival and mortality for many animals simultaneously. Common, pulsed VHF can only monitor one individual on each radio frequency, and the number of individuals monitored is constrained by the amount of time spent on each frequency to facilitate a detection and the number of receivers. Coded VHF largely removes these constraints by using a digital code that can simultaneously monitor up to 512 individuals on a single frequency. Incorporated into an autonomous monitoring system, the coded VHF system also greatly reduces time in the field to confirm the status of individuals. Here we demonstrate the utility of coded VHF technologies applied to monitoring a reintroduced population of brush‐tailed bettong (*Bettongia penicillata*) on the Southern Yorke Peninsula in southern Australia. A system of autonomous monitoring towers was able to monitor 28 different individuals simultaneously without having to change frequency on any of the towers. During a single 24‐h period, one individual was recorded 24,078 times. Key benefits of the high detection rate and autonomous recording are, a timely response to mortalities or a predation event, the detection of nocturnal, cryptic, or burrowing species whenever they are active, and the reduced need for personnel to be in the field.

## Graphical abstract

Using coded VHF technology and autonomous towers to successfully monitor 28 reintroduced Brush‐tailed bettongs (*Bettongia penicillata*). One animal was recorded 24,078 times within a 24 h period and all animals provided daily mortality information in the 2‐week study window and continuously over the next several months.

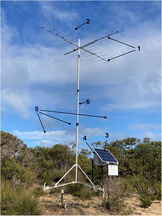

## 1 INTRODUCTION

The relatively new field of reintroduction biology research is dominated by studies on mammals and birds translocated to conserve species and restore ecosystems (Armstrong et al., 2015). Historically many reintroduction attempts have failed, and the practice of reintroduction biology has increasingly emphasized the need to monitor survival outcomes (Moseby et al., 2011; Seddon et al., 2007; Taylor, Canessa, et al., 2017). At the population level, key questions relate to the establishment and persistence of the founder population, including patterns of survival and dispersal during the establishment phase, and the environmental conditions that enable a population to persist in a particular habitat (Armstrong & Seddon, 2008; Moseby et al., 2011). To date, the primary means of monitoring terrestrial mammal populations during translocations has been to tag some or all the released animals with very high frequency (VHF) radio tags, emitting a pulsed signal on individual frequencies. VHF tags (collars, backpacks, tail tags, or other configuration) can provide mortality information and location/movement of animals while being for the most part non‐invasive after deployment. As the scale and scope of any project requiring animal monitoring increases, surveying additional pulsed VHF tags becomes laborious and the data have low temporal and spatial resolution (Thomas et al., 2011). Most receivers can only detect one frequency at a time and therefore, in the case of pulsed VHF technology, one individual at a time (Kays et al., 2011). When monitoring manually, a considerable effort is needed to detect all individuals, particularly in areas of strong signal attenuation as occurs in thick vegetation or mountainous terrain (Fedak et al., 2002) or if the animals are nocturnal and/or use burrows. Burrowing species are mostly not detectable unless active on, or near, the surface (Finlayson et al., 2005). There have been efforts to automate the detection of pulsed VHF tags (Fedak et al., 2002; Gottwald et al., 2019; Griffin et al., 2020; Kays et al., 2011) but the technology remains constrained by the fact that each individual requires a unique frequency. This creates the possibility that an individual can be missed because the receiver was searching a different frequency at the time the animal was detectable.

Digitally encoded radio transmitters or “coded VHF” technology can detect many individuals on the same frequency using a unique digital code for the individual tags. For most reintroduction projects, this means that all individuals are potentially visible to the receiver whenever they are in range, even if only briefly. Thus, animals can be monitored 24/7 using data loggers at base stations with a suitable antenna, while utilizing towers to increase detection range. Coded VHF technology was first developed in the 1990s to monitor fish stocks as they moved through structures such as fish ladders (Hockersmith & Beeman, 2012). In 2012 the technology was further developed to be deployed on birds through the Motus Wildlife Tracking System (https://motus.org) implemented by Birds Canada (Taylor, Crewe, et al., 2017). Avian tags are necessarily small (0.15 g) but needed a large detection range (~15 km) to be detected from fixed towers on their migrations (Taylor, Crewe, et al., 2017). Then in 2018, in collaboration with one of the authors (DR), Lotek (DH and colleagues) developed the first coded VHF radio collars optimized for deployment on terrestrial mammals and the first tower network was constructed the following year by Australian Wildlife Conservancy at Newhaven Wildlife Sanctuary in the Northern Territory, Australia.

In this paper we provide the first report on the utility of coded VHF technology for monitoring terrestrial mammals and the first account of its use for monitoring a mammalian reintroduction in a rewilding project, Marna Banggara (https://www.marnabanggara.com.au), on the Southern Yorke Peninsula (SYP), South Australia. Internationally, there is increasing wildlife welfare and regulatory requirements to monitor reintroduced animals and to report on the fate of individuals. While every translocation has its own unique set of constraints and circumstances, the infrastructure and experiences described in this study will inform others about this new technology, and the advances of coded VHF technology over long‐established pulsed VHF technology and deployments.

## 2 METHODS

### 2.1 Study site

The area of SYP marked for rewilding is bordered by coastal cliffs to the east, south, and west with the northern boundary consisting of a 25 km predator exclusion fence. The area to the west (inside) of the fence comprises approximately 150,000 ha, consisting of ~52% high quality remnant native vegetation (Hodder et al., 2021). It is well suited for a series of reintroductions due to the diversity and extent of remaining habitats, relative to what once existed on SYP; including subcoastal mallee, open and closed woodlands, shrublands on exposed and rocky cliffs, expanses of coastal dunes and shrublands, inland wetlands and saline lakes, as well as swampy forests (Hodder et al., 2021). The general physiognomy of the uncleared vegetation is a dense shrubby understorey of ~0.5 m with a sparse overstory of mostly eucalypt species to ≤10 m in height. In some areas the overstorey forms thickets 3–5 m tall with dense scrub and bush underneath.

**FIGURE 1:**
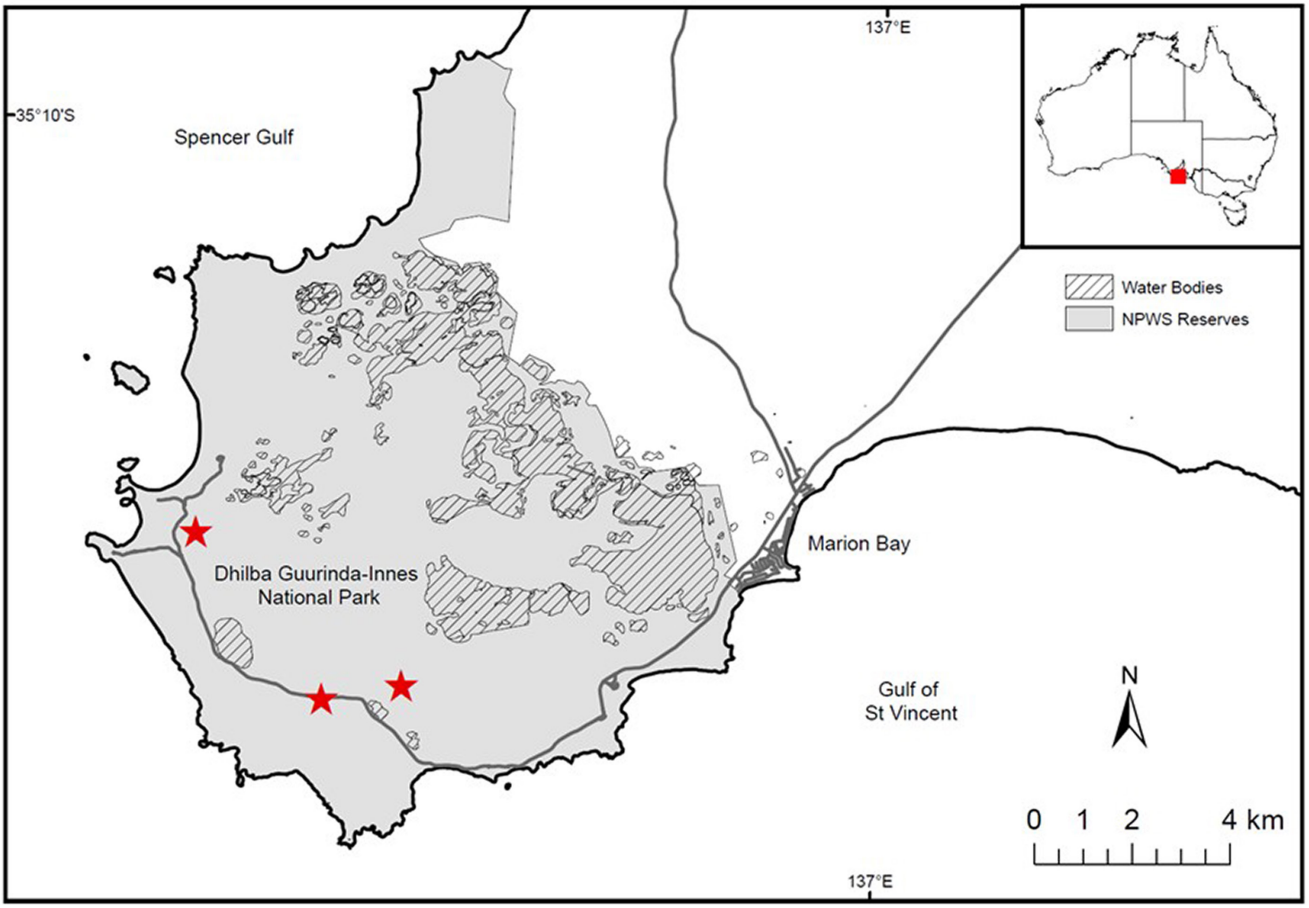
Map showing the tower locations (red star) constructed within of Dhilba Guuranda‐Innes National Park. Mapped water bodies are temporary and dependent of local rainfall and runoff.

An initial release of 40 brush‐tailed bettongs (*Bettongia penicillata*), a critically endangered nocturnal marsupial, were reintroduced to Dhilba Guuranda‐Innes National Park (DGI NP) (35.22S 136.89E) at the southernmost tip of the boot‐shaped SYP (Figure 1). The areas thought to be suitable for the initial release of bettong were sites of high density vegetation, with thick corridors of mallee species (*Eucalyptus diversifolia*, *Eucalyptus rugosa*, and *Eucalyptus porosa*) and tea‐tree (*Melaleuca lanceolata*; Hodder et al., 2021).

### 2.2 Telemetry

#### 2.2.1 Autonomous monitoring towers

**FIGURE 2:**
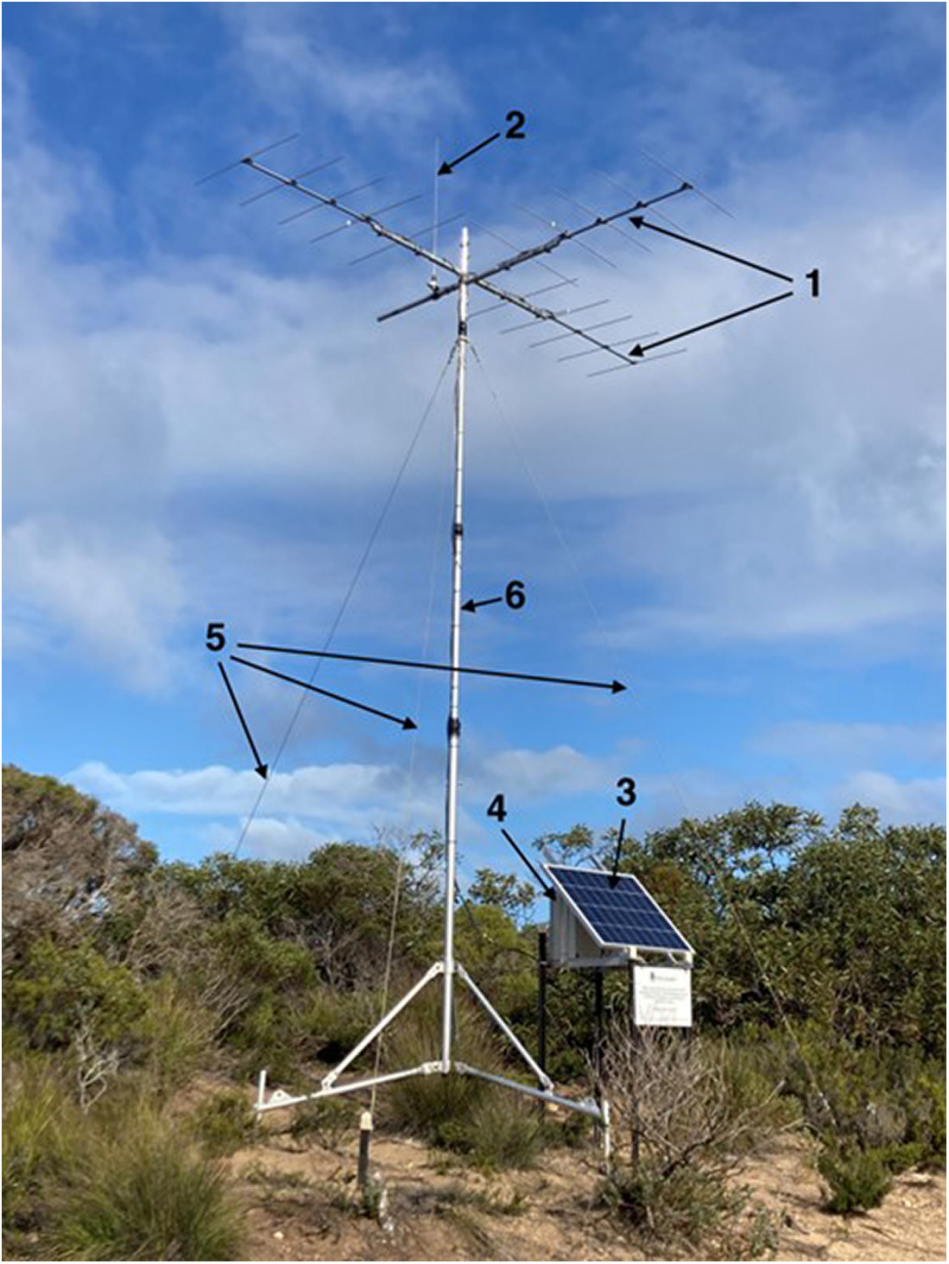
Autonomous monitoring tower (6 m) erected on the Southern Yorke Peninsula, South Australia. Guy wires stabilize the tower in a region subject to high coastal winds. Numbered labels represent key features of the tower mentioned in the text, (1) Yagi antennas, (2) Omni antenna, (3) Solar panel, (4) Locked box containing battery and Lotek SRX1200 Series D datalogger, (5) Guy wires, (6) Telescopic mast.

Three semi‐portable autonomous monitoring stations (Figure 2) were constructed to monitor coded VHF transmitters (collars and tail‐tags) deployed on the reintroduced bettongs. Each station was located at sites chosen for their elevation above sea level and surrounding vegetation, as well as proximity to the release locations. The greater the elevation above the surrounding terrain and vegetation, the less signal attenuation and further the system will reliably detect transmitters. Each site was selected by integrating a digital elevation model (DEM) with algorithms to determine the line‐of‐sight at the height of the antennas on each tower. These were then combined in ArcMAP to determine the likely extent of coverage in the release area and to identify any “blind spots” that may warrant searching if an animal remained undetected (see later).

Towers were fitted with three 5‐element Yagi antennae (Lotek, Canada), and one tuned length omni antenna (Lotek, New Zealand), supported by a 6 m telescopic mast on a three‐legged base secured to the ground with metal pegs and stabilized with three guy wires. The Yagi antennas were orientated to three cardinal points and the omni antenna on the fourth (Figure 2). Orientating the Yagi antenna to the cardinal points facilitated the locating of individuals by examining the relative signal strength on each antenna (see Section 3). The system was powered by a custom‐built solar power unit with an 80 W solar panel and 20 AH deep‐cycle lithium battery (LiFePO4), sufficient to power the receiver through winter at this location. Detection data was stored on a programmable data logger (SRX‐D series, Lotek Canada). The present generation of coded VHF tags and receivers has the capability to simultaneously monitor up to 512 tags on the same frequency, or half that number with mortality indicators. If more capacity is required, the autonomous system can monitor multiple frequencies or combine technologies, monitoring both coded and pulsed VHF tags.

#### 2.2.2 Errors and data cleaning

We removed any unknown or unintelligible IDs (e.g., “999”), which represent interference or anomalies in the data. Less readily identified errors are due to code shift (23, may appear as 24 or 25). This type of error (shift error) occurs more frequently as signal gain is increased on the receiver to extend the detection range. These errors can also be identified and removed from the data set as they occur much more infrequently compared to the “real data” that appears more frequently by orders of magnitude (see Birds Canada (2022) for an extended discussion of data management).

#### 2.2.3 Testing of signal strength and coverage in the release area

Once erected, the towers were tested using two coded VHF collars (Lotek, New Zealand), prior to the reintroduction of animals. Researchers tested collar signal strength at “bettong height” (10 cm from ground), and 160 cm from ground, at 200 m intervals across representative terrain up to 10 km from the towers. This provided baseline information on the range and strength of collar detection and the location of any signal attenuation due to terrain or vegetation density. The difference in height of the collar above ground level was included to determine any impact of vegetation on detectability and relative signal strength. In addition, the gain on the receivers was adjusted between 65 and 90 (maximum 100) to find a value that was the best compromise between tag detection and the insertion of false detections, or noise, into the data set. The identification of a suitable gain for long‐term monitoring is location dependent and is affected by the amount of other radio activity in the area and other factors including atmospheric conditions, and geography. In this instance, a gain of 75 was chosen, as a compromise between consistent detection with distance, and “noise” or “interference” at this coastal location. Having set the basic operating parameters, to test the 24‐h monitoring capabilities and further examine signal attenuation, two collars were deployed at different heights (ground level and 50 cm above ground) in the same location approximately 1 km from the nearest telemetry tower and monitored for 6 days.

#### 2.2.4 Coded VHF tags on translocated animals

The coded VHF collars (Lotek, New Zealand) were used on 28 of the 40 bettongs. The bettongs were released at two sites, 6 km apart, in the study area. The collars were equipped with a whip antenna (20 cm). In previous studies, deployments of VHF collars on brush‐tailed bettongs have used an in‐built brass collar with no external antenna (Priddel & Wheeler, 2004). We chose to use the external whip antenna to improve detection range. Throughout the collar deployment, no issues occurred due to the external antenna.

The autonomous tower system continuously logged the presence and mortality status (alive/dead) of all collared bettongs within range of the towers. A signal record was taken every 5.5 s, this was programmed and predetermined by the design of the tags for this project. The digital signal for each tag is a unique series of peaks and dips with a known duration or “burst rate” (see Taylor, Crewe, et al., 2017 for additional detail). Presence was logged with the collar identification number (ID), antenna number, and relative signal strength index (RSSI) which has a value range up to 250. To incorporate a mortality signal, all individuals had an odd ID and the tag switches automatically to the next even ID if the tag did not move for a period of 4 h. Thus, all even IDs are tags that are not moving (dead animals or dropped tags), if a stationary tag begins to move again it will reactivate to the odd ID. Initially the data was downloaded daily to determine fates of individuals in this first release of animals. As confidence in the system increased, and animals became established in the study area, data download frequency reduced to weekly, fortnightly, then monthly—in accordance with approved translocation protocol.

## 3 RESULTS

### 3.1 Static collar testing

**FIGURE 3:**
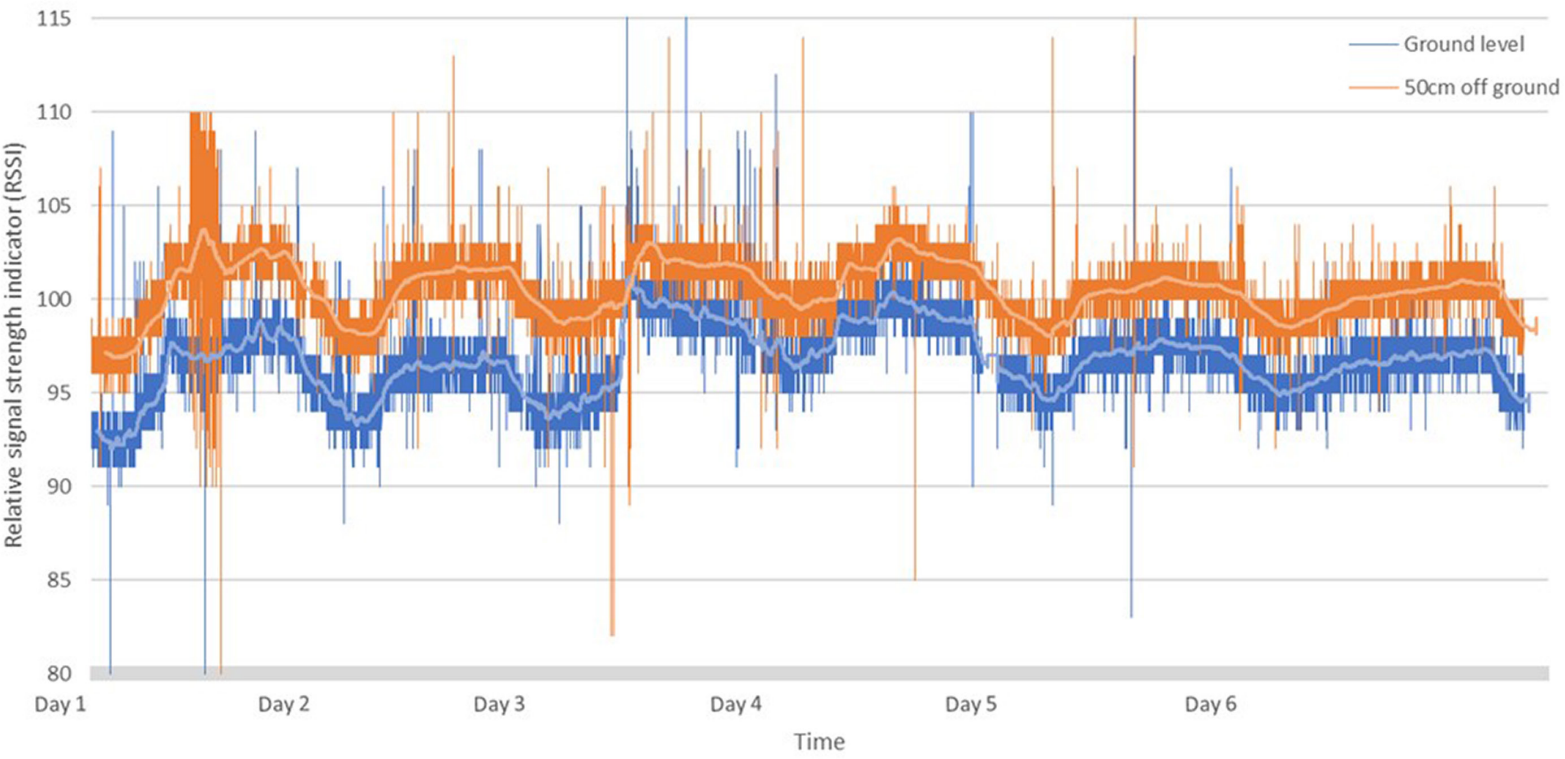
Graphical representation of the RSSI (relative signal strength indication) detected from two stationary coded VHF test collars recorded at a distance of 1 km over 6 days. The blue tracing, is data gathered from collar one, positioned at ground level and orange tracing, data from collar two, positioned 50 cm directly above collar one.

Both static collars were detected at a high frequency, with only a minor difference in detection rates between the two collars placed at different heights. At ground level, the collar was detected on one tower from a single antenna 26,466 times over the six‐day test period, with the higher collar detected 26,912 times. The higher collar (50 cm) was detected at a higher average signal strength, RSSI 101, than the collar at ground level in the same location, RSSI 96. Signal strength was remarkably consistent across the 6 days, and between the collars, although there was a notable periodicity in the data as average RSSI was marginally higher at night than during the day (Figure 3).

### 3.2 Detection of deployed collars and confirmation of animal mortality status

Following release at dusk on 27/08/2021, all 28 collared bettongs were detected the following morning in data collected from the autonomous monitoring towers. Over the next 2 weeks of monitoring before the collars were removed, there were only five instances where an animal was not detected during a 24‐h period. These animals were subsequently detected from multiple towers. It is assumed that any non‐detection was because the animal left the monitoring area covered by the towers and then re‐entered, or was in a location in the study area that was not visible to the towers.

Data download to check for mortality signals took about 30 s to complete per tower for all animals. The autonomous system was able to detect distant tags frequently and consistently. This was confirmed by an animal detected by the tower at a peak RSSI of ~140 at a gain of 75. Standing at the same point, using a handheld Yagi and Lotek SRX1200‐M Series receiver, a researcher was unable to detect the tag manually. Using directional data from the tower (based on signal strength from the antennas) this animal was located 6.5 km away when tracked to its den site using the handheld receiver. The handheld system began to reliably detect the tag at approximately 1.5 km from the den site.

### 3.3 Data density

#### 3.3.1 Daily data collected

**FIGURE 4:**
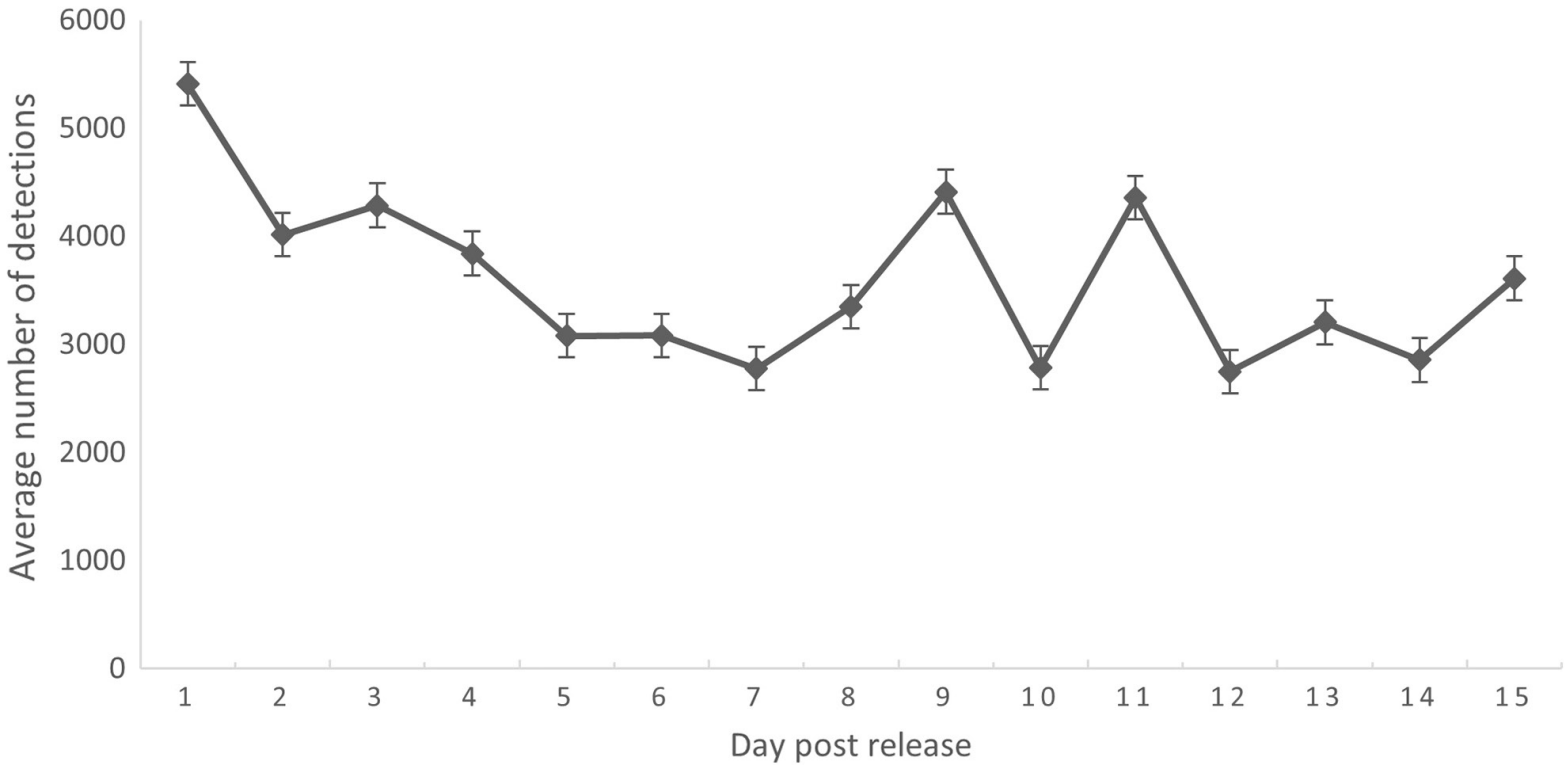
The average number of daily detections of brush‐tailed bettongs (*Bettongia penicillata*) released at DGI NP across all telemetry towers for the first 15 days post release. Presented in a scatter plot with standard deviation. Twenty eight animals were monitored from days 1 to 5 and 27 animals were monitored from days 6 to 15.

The number of times per day that each collar was successfully detected varied day to day with individual animals (Table 1), but remained on average >3000 for all tags (Figure 4). The total number of daily detections across all 28 animals was highest immediately post release, 151,521 tag detections on Day 1, and declined to a minimum of 76,953 tag detections on Day 12 (noting that one animal died on Day 5) before increasing again. As the days post release increased, and the bettongs become settled in their new environment, detection patterns emerge for individuals with some individuals detected more frequently than others. At the initial release of tagged animals, a single tower with 16 collars within its range recorded over 50,000 collar detections in a 24‐h period. A snapshot of the data recorded is presented in Appendix 1.

**TABLE 1:** The number of daily detections of 28 brush‐tailed bettongs (*Bettongia penicillata*) released at DGI NP across all telemetry towers for the first 15 days post release.

Collar	Day 1	Day 2	Day 3	Day 4	Day 5	Day 6	Day 7	Day 8	Day 9	Day 10	Day 11	Day 12	Day 13	Day 14	Day 15
1	521	7	10	1169	737	618	695	483	904	1510	153	0	49	54	60
2	550	500	135	202	6168	1475	1283	2084	2815	919	2235	996	1659	1336	1551
3	24,078	8346	12,432	12,526	9149	9352	9324	10,076	13,897	7279	13,832	10,145	9700	11,309	12,870
4	8604	6544	1535	1486	1196	924	782	1473	2226	30	0	0	0	740	605
5	4695	3166	1330	1964	1752	1666	2409	1533	1316	1350	1142	1165	1291	799	1265
6	999	1094	1131	954	1016	506	21	1688	1765	664	614	599	374	144	230
7	1933	2929	2963	2269	331	683	850	695	3513	2902	3211	214	60	2000	1522
8	3824	1801	2641	3088	2581	2528	2967	3317	4051	2728	3493	2322	4067	3727	4715
9	16,028	8140	12,889	13,663	7594	8771	8697	8712	13,482	7377	13,129	9772	8330	8726	13,139
10	9565	2081	4640	3653	1673	1619	1129	2423	1562	972	1098	1109	669	751	156
11	4354	4893	6067	5318	3560	5566	4797	4635	7481	5612	8703	4670	7733	4933	5391
12	3295	9283	7230	5636	5230	5180	6006	6351	8499	4924	5588	3931	6248	3889	6311
13	2390	102	755	659	682	854	1046	1726	1962	1430	3082	1835	1451	1261	1440
14	640	11,640	9028	8400	5992	6732	6086	6733	8047	6320	8388	7114	12,356	8795	13,415
15	7027	3049	3142	3487	4132	2529	1934	2483	4123	3035	5167	3443	4446	4615	6306
16	3810	733	644	551	1016	651	934	536	1296	698	439	485	526	484	1217
17	3554	9468	10,952	3828	5630	7298	5725	5058	6792	5337	7125	6166	8026	4697	7639
18	4503	7432	8118	5612	5392	7551	476	9913	11,282	5434	8211	5918	9048	5297	243
19	1604	882	2036	1381	2022	2519	3244	3789	5323	2232	6460	3542	2767	2236	4112
20	655	5483	4768	2295	1083	1862	1694	1575	951	1455	1104	608	483	704	1397
21	8986	3061	3678	3197	828	34	99	67	36	150	67	425	1126	1342	1279
22	475	2549	4250	4408	2124	2279	2614	3256	3388	1976	3576	2888	2694	3163	4395
23	13,031	4114	8203	7151	4403	5601	5388	5053	4551	4808	7887	5257	4396	6530	8746
24	13,016	4770	0	6277	6115	6693	5887	8021	11,721	5411	11,156	1769	710	123	70
25	1844	406	1108	324	331	223	373	536	773	587	1433	884	234	611	960
26	270	1649	1289	730	1822	1786	2678	1187	1160	1682	3088	1065	910	1105	1528
27	5747	6819	7967	6737	Mortality	0	0	0	0	0	0	0	0	0	0
28	5523	1482	1083	616	609	825	609	374	653	1118	1641	631	419	621	575

*Note*: Collar number and day 1–15 are indicated. The day with the highest number of detections for each animal has been highlighted in green, and the five instances of 0 daily detections has been highlighted in pink. Data are greyed out post motality.

#### 3.3.2 Recovering collars following a mortality

On day 5 post release, a collar mortality signal was identified (see Table 1). The tower and antenna recording the strongest RSSI informed direction and distance from the tower. A handheld Yagi and portable coded VHF receiver was programmed to search for only the collar mortality ID to be recovered, excluding signals from the other 27 collars on the same frequency. Once the direction and distance were estimated from the tower, it took ~15 min to locate the deceased animal using the handheld tracking equipment. This was done by monitoring the RSSI, its direction, and by reducing the signal gain as the RSSI increased. When using the receiver at the lowest gain (5), signal strength reached 250 when the operator was within 2–3 m of the transmitter.

## 4 DISCUSSION

### 4.1 Benefits of an autonomous, high output, data rich system

The deployment of coded VHF collars and the use of autonomous monitoring towers in this study enabled all 28 tagged bettongs to be detected thousands of times daily within the study area, resulting in substantial data volumes for a low investment in time and manpower. The detection data volumes from each collar where orders of magnitude greater than that typical of studies reliant on pulsed VHF and manual detection of each animal. The high collar detection rate in the first 15 days meant that it was not necessary for additional effort in the field to detect and locate individual animals that were undetected by the towers and dataloggers.

Reintroduction of threatened wildlife occurs across a vast array of landscapes across the world (Armstrong & Seddon, 2008; Seddon et al., 2007). For telemetry, each landscape and situation in which animals occur will come with its own limitations and difficulties. A recurring constraint in the study area was signal attenuation due to dense vegetation and undulating terrain (see Gottwald et al., 2019). Our study indicated that when stationary tags were tested at different heights this affected both collar detection rate and RSSI (Figure 3). The higher collar was detected more often and at a greater signal strength, however, this constraint is mitigated by the high frequency of detection. For brush‐tailed bettongs, that are nocturnal and occupy nests deep in the vegetation during the day, the data volumes from the autonomous towers negated the need to be in the field after dark and mitigated the observed signal attenuation in dense vegetation. The same mitigation of effort in the field would apply to cryptic or burrowing species, perhaps even more so. The automated collection of large volumes of data increases the likelihood that cryptic or burrowing species will be detected during the period they are active and within range.

In the first 15 days post release, only 3 of 28 bettongs were not recorded every day and these animals were only missing from the data for 1–3 days (Table 1). In practice, there only need be a brief amount of time when animals were above ground and visible (i.e., in a less densely vegetated area) for the towers to record their presence, approximate location, and confirm their survival. In contrast, when using pulsed VHF, these events need to be aligned in time and space, including the appropriate frequency being scanned and the observer looking for the animal in the correct location. The coded VHF telemetry system thus removes the possibility of animals being overlooked due to them being subterranean for much of the day or missed because the operator was searching on a different frequency. As data volumes increase, and spatial and temporal coverage becomes more or less complete, the data can be used to model behavioral aspects of the reintroduced population (e.g., Gottwald et al., 2022).

### 4.2 Mobile monitoring systems

At the outset, as the tower signal detection range in the release area was unknown, a contingency plan was devised to ensure sufficient coverage to detect all released animals in this landscape. This involved setting up a Lotek SRX‐M receiver for use with a vehicle, including an omni antenna on the roof attached by a magnetic base. The Lotek SRX‐M receiver was used with a proprietary GPS to log the location of the vehicle with every detection. This data was then used to locate or recover collared animals by providing a start location to track on‐foot with a Yagi and receiver by following the direction of highest signal strength. In this manner, the handheld system could be used for support in locating missing animals, track animals to den sites, or recover animals/collars that had switched to mortality signal. In practice, in this landscape, the mobile system was only used to locate and recover deceased animals as the data recorded by the autonomous towers was sufficient to confirm the status of all tagged animals.

### 4.3 Mortality recognition and response times

Survivorship and the causes of mortality are key performance indicators associated with the success of translocations, and guide management responses. The time between mortality, the recognition of the event, and locating the animal influence the accuracy of any diagnosis of the fate of individuals. In this study, animals were reintroduced to an environment where invasive predators were suppressed, but still active in the landscape. Early recognition of predator related mortality was paramount to enact an on‐ground predator response plan. Recognizing and responding to mortalities in a timely fashion is critical for autopsy and accurate diagnosis of the cause of death. It also provides more accurate indications of an animal's health prior to death, facilitating the collection of higher quality biological samples (microbiome, blood etc). In practice, the continuous collection of collar information via the towers and dataloggers allowed for a same day response to mortality events. The use of remote download via the GSM network would further improve response times. In the case of the single mortality in this study, it took researchers less than an hour from identification of a mortality to locate the animal and collect samples. For this study, the mortality signal is activated after 4 h of no movement. Thus, this system also provides information on a time of death as the collars are continuously monitored.

The towers and coded VHF data loggers have, at the time of writing, been active for 20 months and have provided reliable monitoring of all tagged bettongs in four releases of animals fitted with collars or tail tags. Coded VHF technology provides a reliable, data rich, autonomous system that will facilitate better outcomes for monitoring and research in large multi‐species reintroduction projects.

## AUTHOR CONTRIBUTIONS

**Chloe H. Frick:** Conceptualization (equal); data curation (equal); formal analysis (equal); funding acquisition (equal); investigation (equal); methodology (equal); project administration (equal); writing – original draft (equal); writing – review and editing (equal). **Donell Hole:** Conceptualization (equal); funding acquisition (equal); writing – review and editing (equal). **Derek Sandow:** Investigation (equal); project administration (supporting); writing – review and editing (equal). **Liberty G. M. Olds:** Investigation (equal); project administration (equal); writing – review and editing (equal). **Bertram Ostendorf:** Project administration (equal); writing – review and editing (equal). **David Taggart:** Funding acquisition (equal); investigation (equal); project administration (equal); supervision (equal); writing – review and editing (equal). **David Roshier:** Conceptualization (equal); funding acquisition (equal); methodology (equal); supervision (equal); writing – review and editing (equal).

## Data Availability

Data from the first fortnight of all animals and infield collar tests uploaded online as: Dryad: https://doi.org/10.5061/dryad.h44j0zpq7.

## APPENDIX 1

Example of data set produced by a datalogger and autonomous tower VHF monitoring system collected during the monitoring.
